# Could Obesity Be Related to the Increasing Incidence of Warthin Tumors?

**DOI:** 10.3390/jcm13164935

**Published:** 2024-08-21

**Authors:** Michał Gontarz, Jakub Bargiel, Krzysztof Gąsiorowski, Tomasz Marecik, Paweł Szczurowski, Andrei Hramyka, Joanna Kuczera, Agata Wieczorkiewicz, Grażyna Wyszyńska-Pawelec

**Affiliations:** 1 Department of Cranio-Maxillofacial Surgery, Jagiellonian University Medical College, 30-688 Cracow, Poland; jakub.bargiel@uj.edu.pl (J.B.); krzysztof.gasiorowski@uj.edu.pl (K.G.); tomasz.marecik@uj.edu.pl (T.M.); pawel.szczurowski@uj.edu.pl (P.S.); grazyna.wyszynska-pawelec@uj.edu.pl (G.W.-P.); 2 Students’ Scientific Group of the Department of Cranio-Maxillofacial Surgery, Jagiellonian University Medical College, 30-688 Cracow, Poland; andrei.hramyka@student.uj.edu.pl (A.H.); joanna.kuczera@student.uj.edu.pl (J.K.); a.wieczorkiewicz@student.uj.edu.pl (A.W.)

**Keywords:** Warthin tumor, adenolymphoma, obesity, aging, cigarette smoking, chronic inflammation, etiology

## Abstract

**Background**: The number of patients diagnosed with Warthin tumors (WTs) has increased significantly in recent years. The association of obesity as measured by body mass index (BMI) with the incidence of WTs remains unclear. This retrospective study aims to compare the BMI and other clinical factors of patients diagnosed with WTs to those with other benign epithelial parotid gland tumors. **Methods**: Over a 24-year period, 465 cases of benign epithelial parotid gland tumors were treated in our department. Of these, 155 (33.3%) were diagnosed as WTs. The results of the WT group were compared with those of another benign epithelial parotid gland tumor. **Results**: The mean BMI of WT patients was 27.3, which was significantly higher than in other benign tumors (25.52; *p* < 0.001). Furthermore, statistically significant correlations were observed, including a higher incidence of WT in males (*p* < 0.001), in the elderly (*p* < 0.001), and in cigarette smokers (*p* < 0.001). Additionally, a higher prevalence of other head and neck cancers was confirmed in patients with WTs (*p* = 0.004); **Conclusions**: This study supports the multifactorial etiology of WT development. Among these factors, smoking, advanced age, and obesity have been identified as factors associated with the development of WT, which might be due to chronic inflammation linked to obesity.

## 1. Introduction

Adenolymphoma, cystadenolymphoma, or papillary cystadenoma lymphomatosum was first described in 1895 by Hildebrand as a congenital cyst of the neck [1]. The term “papillary cystadenoma” was subsequently proposed by Albrecht and Artz in 1910 [2]. In 1929, American pathologist Aldred Scott Warthin described two additional cases in the English literature and introduced the term “papillary cystadenoma lymphomatosum” [3]. Since then, this tumor has been known as Warthin’s tumor (WT). WT is the second most common benign tumor of the salivary glands. WT is most commonly observed in the tail of the parotid gland. An extraparotid manifestation is uncommon and frequently involves nodes proximate to the parotid gland, particularly in neck level II [4,5].

Microscopically, WTs are composed of two components that vary in proportion from case to case. The first component is the true neoplastic oncocytic bilayered epithelium, which grows in cystic, solid, and papillary patterns. The second component is reactive stromal lymphoid tissue, often with lymph follicles present [6]. The epithelial and lymphoid components of WT may undergo malignant transformation, although this is a rare entity [7]. Additionally, the lymphoid components of WT may serve as a site for tumor-to-tumor regional and distant metastasis [8,9].

The number of patients diagnosed with WT has increased significantly in recent years [10,11,12]. Cigarette smoking is the only confirmed risk factor for the development of WT. Other explanations for the rise in incidence could be longer lifespan (senescent cells), improved diagnostic tools and imaging sensitivity, radiation exposure, chronic inflammation, metabolic dysfunction of mitochondria and Epstein–Barr virus, human papilloma virus (HPV) infection, or IgG4-related disease (IgG4-RD) [6,13,14]. In patients with oncological conditions, particularly in the context of PET/CT imaging, WT may be identified as an incidental tumor. Conversely, as evidenced by the study by Kadletz et al., the elevated prevalence of obesity may also be associated with a higher incidence of WT [15]. However, the association between high body mass index (BMI) and WT risk has not been confirmed by other reports in the literature. Furthermore, there is a lack of a multivariable analysis of BMI in comparison to other clinical factors influencing the incidence of WT.

This retrospective study aims to compare the BMI and other clinical factors of patients diagnosed with WT to those with other benign epithelial parotid gland tumors.

## 2. Materials and Methods

Between January 2000 to December 2023, 668 patients were treated for parotid gland tumors in the Department of Cranio-Maxillofacial Surgery of the Jagiellonian University in Cracow.

**Figure 1:**
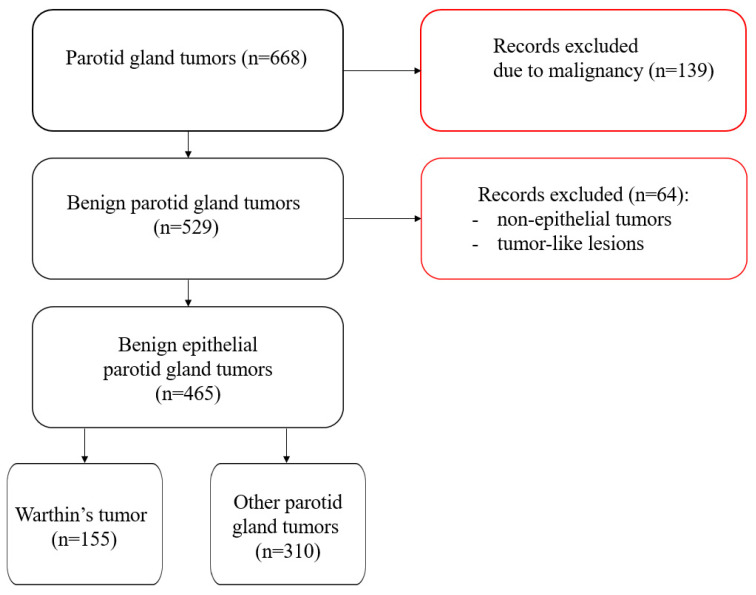
Flow chart of the study population.

Patients with benign epithelial tumors of the parotid gland were included in this study. Patients operated upon due to malignant tumors—such as salivary gland cancers, lymphomas or metastases to the parotid gland—were excluded from the study. Additionally, benign nonepithelial tumors (lipoma, hemangioma, Schwannoma) and tumor-like lesions of the parotid gland (cysts, chronic inflammations, benign lymphoepithelial lesion, IgG4-related disease, sclerosing polycystic adenosis, Sjögren’s syndrome, tuberculosis, oncocytic metaplasia, cat scratch disease, and toxoplasmosis) were excluded (Figure 1).

The tumors were benign epithelial salivary gland neoplasms in 465 cases, 155 (33.3%) of which were diagnosed as WT. The medical charts of the patients were evaluated according to demographic characteristics, histopathological aspects, diameter and site of the tumor, smoking habits, BMI, and synchronous and metachronous tumors in head and neck region. BMI was assessed secondarily using the patient’s weight and height recorded on admission to the department prior to planned surgical treatment. The results of the WT group were compared with those of other benign epithelial parotid gland tumors, and the differences in terms of multifocal, bilateral, and extraparotid WTs were also assessed.

This study was approved by the institutional review board. As only medical files were obtained, the review board approved this study without the need for patient consent as long as all personal information was kept confidential and any facial features or other identifying marks were removed and/or covered.

Comparison of the values of qualitative variables within groups was performed using the chi-square test (with Yates adjustment for 2 × 2 tables) or Fisher’s exact test when the assumptions of the chi-square test regarding expected numbers were not met. Comparisons of quantitative variables in the two groups were made using the Mann–Whitney test. Multivariable analysis of the effect of potential predictors on a dichotomous variable (i.e., taking only two possible values—WT or other tumors) was performed using logistic regression. The results were rearranged in the form of OR (odds ratio) parameters along with 95% confidence intervals. The analysis assumed a significance level of 0.05. Thus, all *p*-values below 0.05 were interpreted as indicating significant relationships. The analysis was performed using R software, version 4.4.0.

## 3. Results

### 3.1. WT vs. Epithelial Benign Parotid Tumors

Table 1 shows the differences between the groups of patients with WT and other epithelial benign parotid tumors. Statistically significant correlations were observed, such as a higher incidence of WT in males (*p* < 0.001), the elderly (*p* < 0.001), and cigarette smokers (*p* < 0.001). A higher prevalence of other head and neck cancers (*p* = 0.004) and higher BMI (*p* < 0.001) was also confirmed in patients with WT.

In the multivariable logistic regression analysis of patients with benign parotid tumors (Table 2), it was found that

- each additional year of age increases the odds of a WT by 9.4% (OR = 1.094);
- female gender decreases the odds of WT by 59.5% (OR = 0.405) compared to male gender;
- presence of another head and neck cancer increases the odds of WT by 5.713 times (OR = 5.713);
- each additional kg/m^2^ in BMI increases the odds of WT by 12.1% (OR = 1.121);
- smoking up to 10 cigarettes, 10–20 cigarettes, and more than 20 cigarettes per day increases the odds of WT by 5.136 (OR = 5.136), 14.014 (OR = 14.014) and 34.526 times (OR = 34.526), respectively.

### 3.2. Unifocal vs. Multifocal Parotid WT

Analysis of the relationship between patients with single WT and multiple WT showed no statistically significant relationship. BMI was comparable in both groups (*p* = 0.497) (Table 3).

### 3.3. Unilateral vs. Bilateral WT

Comparison of the group of patients with bilateral and unilateral WT revealed a statistically significant higher percentage of patients who smoked more and had bilateral tumors (*p* = 0.038). BMI was also comparable in both groups (*p* = 0.786) (Table 4).

### 3.4. Extraparotid vs. Intraparotid WT

**Figure 2:**
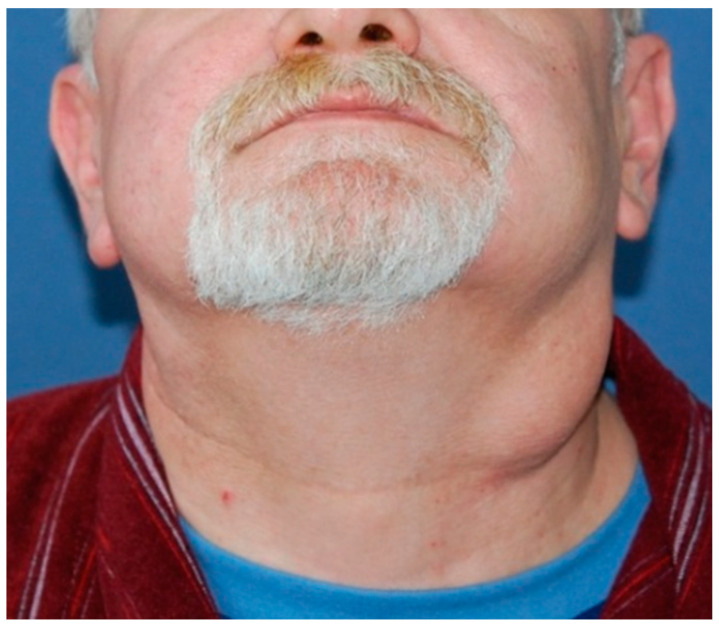
Clinical view of a patient with bilateral WTs and BMI 35.5 kg/m^2^ who smokes more than 20 cigarettes per day. Extraparotid localization in cervical levels II and III is visible on the left side.

In eight cases, extraparotid WT was found in cervical lymph node levels II and III (Figure 2). When comparing the groups of patients with parotid and extraparotid localization, there were also no statistically significant differences between these groups (Table 5). However, there was an apparent trend toward a higher incidence of extraparotid WT in the obese group (*p* = 0.08) and at a younger age (*p* = 0.095).

## 4. Discussion

Obesity is a chronic, progressive, and recurrent condition that leads to numerous adverse health, psychological, social, and economic consequences. According to the World Obesity Federation Annual Report 2020, the global prevalence of obesity is projected to increase from 11.4% in 2010 to 17.5% by 2030, with over 1 billion people expected to be living with obesity by then. At that time, one in five women and one in seven men will be obese [16]. Additionally, life expectancy is predicted to decrease by 0.9–4.2 years in the Organization for Economic Cooperation and Development (OECD), Group 20 (G20), and European Union (EU28) countries over the next 30 years [17].

Adipose tissue cells secrete adipokines and cytokines, which attract immune cells and promote a pro-inflammatory condition. Obesity-associated inflammation is first triggered by excess nutrients and is primarily localized in specialized metabolic tissues such as white adipose tissue. However, this low-grade inflammatory response associated with obesity leads to changes in immune cell infiltration and polarization not only in white adipose tissue but also in other organs, resulting in systemic inflammation [18,19,20]. Obesity also activates numerous oncogenic signaling pathways that promote tumor cell survival, proliferation, and metabolism. It is also worth noting that inflammation associated with obesity can be found in the salivary glands. Lehmann et al. observed an increased level of inflammatory markers in saliva in obese patients, including tumor necrosis factor-α receptors 1 and 2 (TNF-α-R1, TNF-α-R2), pentraxin 3 (PTX-3), interleukin 15 (IL-15), monocyte chemoattractant protein 1 (MCP-1), and soluble intercellular adhesion molecule 1 (slCAM-1) [21]. Some studies have also indicated a potential link between obesity and the occurrence of salivary gland tumors. Suba et al. found that obesity was more prevalent among salivary gland tumor patients than in the control group (*p* < 0.001) [22]. Leopard et al. observed a significantly higher incidence of adenoid cystic carcinoma in areas with increased obesity in the Welsh population (*p* = 0.028) [23]. Forrest et al. in Canada reported that an obese BMI was associated with an almost 40% increased risk of salivary gland cancer [24].

A study by Kuzenko et al. indicated that WT has an inflammatory etiology [6]. Obesity as inflammatory induce factor may be one of the triggers of WT development. Additionally, a reduction in the body’s endogenous antioxidant levels as a consequence of an unhealthy diet may also be a contributing factor. Kadletz et al. showed that WT’s patients had a significantly higher BMI in comparison to patients with other benign parotid gland tumors (29.1 vs. 26.2, *p* < 0.0001) [15]. Similar findings were observed in this study (27.17 vs. 25.62 *p* < 0.001). However, there was no correlation between the multifocal occurrence of WT in one parotid gland or bilateral parotid occurrence, depending on the patient’s BMI. Conversely, there was a trend towards a higher risk of extraparotid WT localization in patients with a higher BMI (*p* = 0.08). This phenomenon can probably be explained by a more intense local inflammatory process taking place in the white adipose tissue of the neck in the course of obesity. Further evidence that nutrition may play a role in the development of WT is provided by the fact that the prevalence of WT in the African population is very low [25]. In some epidemiological studies, for example in Uganda, no cases of WT were observed [26]. The incidence of obesity and overweight in the adult population of Uganda is 9.3% and 14.6%, respectively [27]. In contrast, research by Saravakos et al. has identified WT as the most common salivary gland tumor [10]. This study was conducted in Germany, where the prevalence of obesity and overweight in the adult population is 19% and 34.5%, respectively [27].

A well-documented factor that influences the development of WT is smoking cigarettes [28]. The current study found that an increase in the number of cigarettes smoked was associated with a significantly elevated risk of a parotid gland tumor being WT, with an odds ratio from 5.13 to 34.52. In addition, the correlation between cigarette smoking and the risk of bilateral WT was observed, but not for multifocal WT in one parotid gland. Cigarette smoke increases the production of reactive oxygen species (ROS), including H_2_O_2_ and O_2_-. Among the numerous constituents of cigarette smoke, there are DNA-binding agents that have been shown to cause DNA damage, which tends to accumulate, particularly in the mitochondria [29]. The study by Lewis et al. identified an elevated prevalence of the 4977 bp deletion in the mitochondrial genome in smokers and in the oncocytic tumor cells of the WT population [29].

The question of whether mtDNA damage in WT is solely a direct result of cigarette smoke is open to debate. However, smokers tend to consume diets that are less nutritionally balanced and contain fewer dietary antioxidants, and they also consume more alcohol than non-smokers [30]. The reduction in exogenous antioxidants, in conjunction with cigarette smoke, could significantly contribute to increased oxidative damage to mtDNA and more deletions within the parotid gland. Moreover, the results of an experimental study by Yang et al. on mice indicated that the cigarette smoke exposure could disrupt the homeostasis of cholesterol and bile acid metabolism within the liver and subsequently influence the composition and diversity of the gut microbiota. Furthermore, cigarette smoke caused insulin resistance in the livers of mice on a high-fat diet [31].

**Figure 3:**
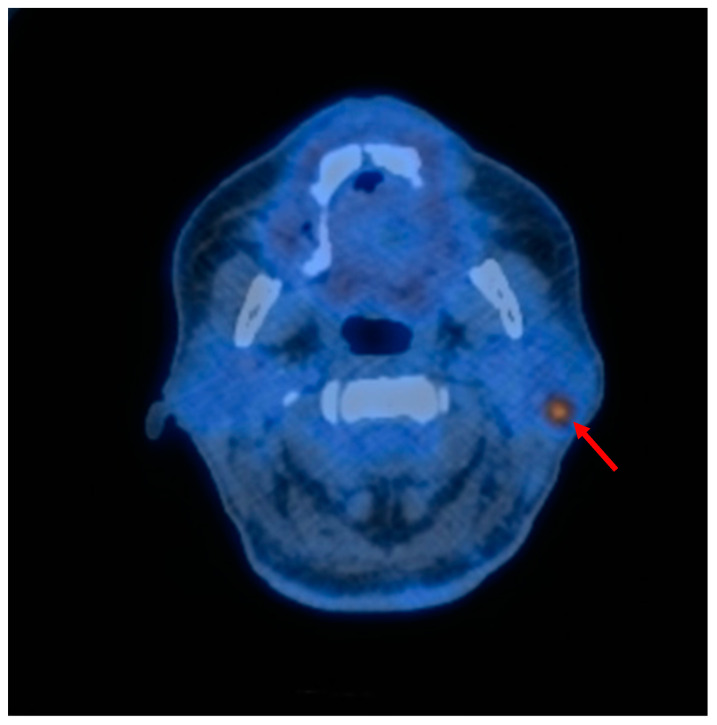
PET/CT scan: horizontal view with WT in the tail of the left parotid gland in a patient following resection of squamous cell carcinoma of the floor of the mouth. Abnormal, high FDG uptake on PET/CT in WT may be a misleading indication of lymph node metastasis (red arrow).

Furthermore, smoking has been linked to an increased risk of other cancers of the head and neck region. This relationship was observed with greater prevalence in the WT group (*p* = 0.004). A total of 13 patients in the WT tumor group exhibited concomitant other cancers. The most prevalent cancers were oral squamous cell carcinomas (OSCCs) (nine cases), skin cancers (four cases), and lip cancer (one case). In one patient, a synchronous tumor-to-tumor metastasis of skin cancer was identified in the WT of the parotid gland [8]. The majority of WTs were identified metachronically during the oncological follow-up of patients with OSCC. Nevertheless, it should be noted that abnormal 18F-fluorodeoxyglucose (FDG) uptake in PET/CT is a typical feature of WTs and may suggest the presence of metastatic lymph nodes in synchronous appearance with OSCC (Figure 3) [5].

Another factor associated with WT appearance was old age of patients (*p* < 0.001). In this study, based on multivariate analysis, each additional year of life increased the odds that a benign tumor located in the parotid gland was a WT by 9.4% (OR = 1.094). The proportion of senescent cells in tissues and organs increases with age, contributing to both the aging process and the development of age-related diseases (ARDs). Despite remaining in the G1 phase of the cell cycle, these cells exhibit active intracellular metabolism, as evidenced by the secretion of proteins such as inflammatory cytokines. The senescence-associated secretory phenotype (SASP) is a phenotype associated with senescent cells that secrete high levels of inflammatory cytokines, immune modulators, growth factors, and proteases. These include interleukin 1β (IL1β), IL6, IL8, and plasminogen activator inhibitor 1 (PAI-1), among others [14,32,33]. Studies have demonstrated that SASP factors can contribute to the development of ARD, promote oncogenesis, and facilitate tumor growth while enhancing tissue repair and regeneration [32,34,35].

The gold standard for the treatment of WT is surgical removal of the tumor from the parotid gland. The extent of the surgical procedure is frequently contingent upon the location, size, and potential multifocality of the lesion. For single tumors classically located in the tail of the parotid gland, extracapsular dissection (ECD) and partial parotidectomy have been demonstrated to provide good treatment results. In contrast, multifocal WTs in the superficial lobe can also be treated by ECD. However, in most cases, due to their close proximity to each other in the parotid gland, they are treated by partial lateral or lateral (superficial) parotidectomy. In the case of multifocal lesions involving both lobes of the parotid gland, total parotidectomy with facial nerve preservation is the recommended treatment [36]. Furthermore, in the event that the tumor is situated in the tail of the parotid gland, in the computed tomography (CT) or magnetic resonance imaging (MRI) scans, the level II neck lymph nodes should be evaluated due to the potential for the presence of foci of extraparotid WTs.

In addition to surgical procedures, there are several non-surgical methods for treating WT. These include microwave ablation (MWA), radiofrequency ablation (RFA), and ultrasound-guided ethanol sclerotherapy (UGES) [36,37,38,39,40].

These non-surgical options demonstrate considerable potential, as evidenced by their effectiveness. They could significantly alter the treatment of WT in the future. However, long-term prospective outcome studies are needed to establish their proper role before they can be widely adopted for WT treatment.

In certain cases of WT, due to the low risk of malignant transformation, which is confirmed by FNAC and the patient’s numerous comorbidities that exclude general anesthesia, a “wait-and-see” policy can be applied. WT is characterized by slow growth, which further decreases after the patient reaches 75 years of age [41]. One marker that assesses the growth of WT is Ki-67. The expression of the Ki-67 proliferation marker, which detects all phases of the cell cycle except G0, is known to predict disease outcome in many human malignancies [6]. In most cases, the Ki-67 labeling index in WT is less than 5%, and its increase may be indicative of malignant transformation [42,43].

## 5. Study Limitations

This study is limited by its retrospective analysis of the clinical features of patients operated on for benign epithelial parotid tumors at a single clinical center. Additionally, our study group is relatively small and has not been compared with the standard population. The next variable that may influence the overall results is the human-performed collection of medical data with BMI analysis and its interpretation. Due to the retrospective nature of this study and the limited data available to assess patients’ rates of metabolic syndrome-associated comorbidities such as lipoprotein (HDL, LDL), total serum cholesterol, triglyceride, glycated haemoglobin, gamma-glutamyl transpeptidase, aspartate transaminase, alanine transaminase, fibrinogen, C-reactive protein and erythrocyte sedimentation rate were not included in the analysis.

Another concern is the use of BMI in this study to assess the potential impact on the development of chronic inflammation. Currently, obesity is defined by an elevated BMI, which is typically a result of excess adipose tissue. Nevertheless, insulin resistance and inflammation have been documented in individuals with a normal BMI. Conversely, a minority of individuals with an elevated BMI are metabolically healthy. Consequently, the current definition of obesity based on BMI has recently been called into question, and BMI may be inadequate for identifying patients with adipose tissue inflammation [44].

A future prospective study with multivariate analysis assessing the impact of laboratory tests involved in the pro-inflammatory cascade in relation to BMI, nutritional status, and dietary intake would increase the reliability of the evidence for an inflammatory etiology of WT.

## 6. Conclusions

**Figure 4:**
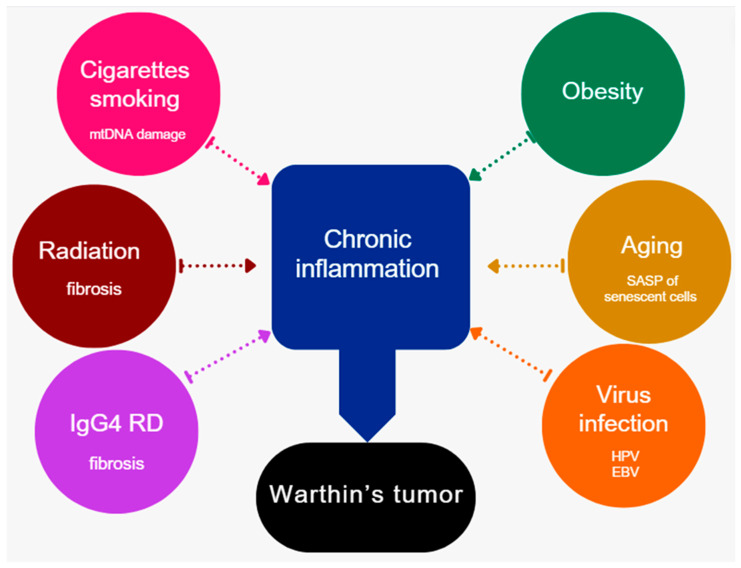
Graphic summarizing the multifactorial etiology of developing WT.

This study supports the multifactorial etiology of WT development. Among these factors, smoking, advanced age, and obesity have been identified as contributing to the development of WT potentially due to chronic inflammation, which is linked to obesity (Figure 4). The chronic inflammation observed in obese patients may exacerbate the risk of WT, as evidenced by the higher levels of obesity in the younger population of patients with WT and the increase in the incidence of this tumor observed in the populations of developed countries. It is also appropriate to categorize this disease as an age-associated disease (ARD) given the significantly higher incidence observed in individuals over the age of 60. This may be a consequence of the SASP of senescent cells, which are known to produce a range of pro-inflammatory cytokines. Furthermore, the incidence of bilateral WTs demonstrated a positive correlation with the quantity of cigarette smoking. However, multifocal WTs in a single parotid gland were not found to be associated with the amount of cigarette smoking.

## Figures and Tables

**Table 1:** Clinical differences between the groups of patients with WT and other epithelial benign parotid tumors.

Parameter		Warthin’s Tumor (*n* = 155)	Other Benign Tumors (*n* = 310)	*p*
BMI [kg/m^2^]	Average (SD)	27.3 (3.84)	25.52 (4.42)	*p* < 0.001
	Median (quartile)	27.17 (24.82–29.61)	25.62 (22.21–27.64)	
	Range	18.34–40.9	16.65–40.51	
	*n*	155	310	
Age (years)	Average (SD)	64.37 (8.75)	48.34 (16.72)	*p* < 0.001
	Median (quartile)	65 (59–70)	49 (36–61)	
	Range	42–87	11–85	
	*n*	155	310	
Sex	Male	99 (63.87%)	111 (35.81%)	*p* < 0.001
	Female	56 (36.13%)	199 (64.19%)	
Diameter of the tumor [mm]	Average (SD)	25.03 (10.67)	27.57 (10.48)	*p* = 0.012
	Median (quartile)	23.5 (18–32)	26 (20–35)	
	Range	4–79	8–65	
	*n*	154	304	
Site of the tumor	Superficial lobe	128 (82.58%)	261 (84.19%)	*p* < 0.001
	Deep lobe	19 (12.26%)	49 (15.81%)	
	Neck lymph nodes	8 (5.16%)	0 (0.00%)	
Multifocal tumor in the parotid gland	No	129 (83.23%)	300 (96.77%)	*p* < 0.001
	Yes	19 (12.26%)	10 (3.23%)	
	No data	7 (4.52%)	0 (0.00%)	
Facial nerve dysfunction	No	140 (90.32%)	260 (83.87%)	*p* = 0.08
	Yes	15 (9.68%)	50 (16.13%)	
Other head and neck cancer	No	141 (90.97%)	303 (97.74%)	*p* = 0.004
	Yes	13 (8.39%)	7 (2.26%)	
	No data	1 (0.65%)	0 (0.00%)	
Cigarette smoking	Non-smoker	26 (16.77%)	238 (76.77%)	*p* < 0.001
	Cigarettes <10/day	25 (16.13%)	44 (14.19%)	
	Cigarettes 10–20/day	46 (29.68%)	17 (5.48%)	
	Cigarettes >20/day	52 (33.55%)	10 (3.23%)	
	Former smoker	6 (3.87%)	1 (0.32%)	

SD—Standard deviation; BMI: Body Mass Index.

**Table 2:** Multivariable logistic regression describing risk factors for WT in a group of patients with benign epithelial parotid tumors.

Feature		OR	95%CI		*p*-Value
BMI (kg/m^2^)		1.121	1.039	1.21	0.003
Age (years)		1.094	1.064	1.125	<0.001
Sex	Male	1	ref.		
	Female	0.405	0.216	0.761	0.005
Tumor diameter (mm)		0.979	0.949	1.01	0.179
Site of the tumor	Superficial lobe	1	ref.		
	Deep lobe	0.568	0.235	1.375	0.21
	Neck lymph nodes	---	---	---	---
Multifocal tumor in the parotid gland	No	1	ref.		
	Yes	3.026	0.789	11.607	0.107
Other head and neck cancer	No	1	ref.		
	Yes	5.713	1.081	30.204	0.04
Cigarette smoking	Non-smoker	1	ref.		
	Cigarettes <10/day	5.136	2.307	11.431	<0.001
	Cigarettes 10–20/day	14.014	5.955	32.975	<0.001
	Cigarettes >20/day	34.526	12.262	97.215	<0.001
	Former smoker	9.336	0.946	92.11	0.056
Duration of symptoms		0.978	0.964	0.991	0.001

ref.—reference level; OR—odd ratio; CI—confidence interval.

**Table 3:** Analysis of the relationship between patients with unifocal WT and multifocal WT in the parotid gland.

Parameter		Multifocal WT in the Parotid Gland		*p*-Value
		No (*n* = 129)	Yes (*n* = 19)	
BMI (kg/m^2^)	Average (SD)	27.33 (3.77)	26.23 (4.16)	*p* = 0.497
	Median (quartile)	27.17 (24.91–29.35)	26.7 (22.46–29.77)	
	Range	18.51–40.9	18.34–32.72	
	*n*	129	19	
Cigarette smoking	Non-smoker	23 (17.83%)	3 (15.79%)	*p* = 0.315
	Cigarettes <10/day	24 (18.60%)	1 (5.26%)	
	Cigarettes 10–20/day	38 (29.46%)	5 (26.32%)	
	Cigarettes >20/day	38 (29.46%)	10 (52.63%)	
	Former smoker	6 (4.65%)	0 (0.00%)	
Age (years)	Average (SD)	64.43 (8.58)	65.68 (7.73)	*p* = 0.269
	Median (quartile)	65 (59–70)	67 (63.5–70)	
	Range	42–87	42–79	
	*n*	129	19	
Sex	Male	82 (63.57%)	13 (68.42%)	*p* = 0.876
	Female	47 (36.43%)	6 (31.58%)	

**Table 4:** Analysis of the relationship between patients with bilateral and unilateral WTs.

Parameter		Unilateral (*n* = 134)	Bilateral (*n* = 21)	*p*-Value
BMI (kg/m^2^)	Average (SD)	27.34 (3.91)	27.02 (3.41)	*p* = 0.786
	Median (quartile)	27.35 (24.82–29.77)	26.83 (25.35–29.32)	
	Range	18.34–40.9	20.28–34.06	
	*n*	134	21	
Cigarette smoking	Non-smoker	25 (18.66%)	1 (4.76%)	*p* = 0.038
	Cigarettes <10/day	24 (17.91%)	1 (4.76%)	
	Cigarettes 10–20/day	41 (30.60%)	5 (23.81%)	
	Cigarettes >20/day	39 (29.10%)	13 (61.90%)	
	Former smoker	5 (3.73%)	1 (4.76%)	
Age (years)	Average (SD)	64.13 (9)	65.86 (7.01)	*p* = 0.584
	Median (quartile)	65 (59–70)	65 (60–67)	
	Range	42–87	58–86	
	*n*	134	21	
Sex	Male	84 (62.69%)	15 (71.43%)	*p* = 0.595
	Female	50 (37.31%)	6 (28.57%)	

**Table 5:** Analysis of the relationship between patients with intraparotid and extraparotid WTs.

Parameter		Parotid WT (*n* = 147)	Extraparotid WT (*n* = 8)	*p*-Value
BMI [kg/m^2^]	Average (SD)	27.17 (3.83)	29.63 (3.5)	*p* = 0.08
	Median (quartile)	27.16 (24.45–29.4)	29.94 (27.76–30.77)	
	Range	18.34–40.9	24.82–35.51	
	*n*	147	8	
Cigarette smoking	Non-smoker	26 (17.69%)	0 (0.00%)	*p* = 0.319
	Cigarettes <10/day	25 (17.01%)	0 (0.00%)	
	Cigarettes 10–20/day	43 (29.25%)	3 (37.50%)	
	Cigarettes >20/day	47 (31.97%)	5 (62.50%)	
	Former smoker	6 (4.08%)	0 (0.00%)	
Age (years)	Average (SD)	64.6 (8.49)	60.12 (12.73)	*p* = 0.095
	Median (quartile)	65 (59.5–70)	58.5 (54–63.5)	
	Range	42–87	42–86	
	*n*	147	8	
Sex	Male	94 (63.95%)	5 (62.50%)	*p* = 1
	Female	53 (36.05%)	3 (37.50%)	

## Data Availability

Restrictions apply to the availability of these data. The data were obtained from patients treated at the Department of Cranio-Maxillofacial Surgery, Cracow, Poland, and cannot be shared in accordance with the General Data Protection Regulation (EU) 2016/679.

## References

[1] Bonanthaya K., Panneerselvam E., Manuel S., Kumar V.V., Rai A. (2021). Oral and Maxillofacial Surgery for the Clinician.

[2] Albrecht H., Arzt L. (1910). Beiträge zur Frage der Gewebsveirrung. Papilläre Cystadenome in Lymphdrüsen. Frankf. Z. Pathol..

[3] Warthin A.S. (1929). Papillary cystadenoma lymphomatosum. A rare teratoid of the parotid gland region. J. Cancer Res..

[4] Xu W., Lu H., Zhu Y., Ruan M., Zhang C., Yang W., Liu S. (2018). Warthin’s tumour in oral and maxillofacial regions: An 18-year retrospective study of 1084 cases in an eastern-Chinese population. Int. J. Oral Maxillofac. Surg..

[5] Gontarz M., Gąsiorowski K., Bargiel J., Marecik T., Szczurowski P., Zapała J., Wyszyńska-Pawelec G. (2021). Extraparotid Warthin Tumors Imitating Metastasis of Oral Cancers. Int. Arch. Otorhinolaryngol..

[6] Kuzenko Y.V., Romanuk A.M., Dyachenko O.O., Hudymenko O. (2016). Pathogenesis of Warthin’s tumors. Interv. Med. Appl. Sci..

[7] Seifert G., Heckmayr M., Donath K. (1977). Carcinome in Papillären Cystadenolymphomen der Parotis Definition und Differentialdiagnose [Carcinomas in papillary cystadenolymphomas of the parotid gland--definition and differential diagnosis (author’s transl)]. Z. Krebsforsch. Klin. Onkol. Cancer Res. Clin. Oncol..

[8] Gontarz M., Gałązka K., Gąsiorowski K., Bargiel J., Marecik T., Szczurowski P., Wyszyńska-Pawelec G. (2024). Tumor-to-Tumor Metastasis: Dissemination of Cutaneous Squamous Cell Carcinoma Involving Parotid Warthin Tumor—Case Report. Diseases.

[9] Laco J., Celakovsky P., Kalfert D., Hornychova H., Rybnikar T., Ryska A. (2010). Tumor-to-tumor metastasis: Warthin tumor as a recipient of lung carcinoma and of renal carcinoma—Report of two cases. Pathol. Res. Pract..

[10] Saravakos P., Kourtidis S., Hartwein J., Preyer S. (2022). Parotid Gland Tumors: A Multicenter Analysis of 1020 Cases. Increasing Incidence of Warthin’s Tumor. Indian J. Otolaryngol. Head Neck Surg..

[11] Franzen A.M., Kaup Franzen C., Guenzel T., Lieder A. (2018). Increased incidence of Warthin tumours of the parotid gland: A 42-year evaluation. Eur. Arch. Otorhinolaryngol..

[12] Gontarz M., Bargiel J., Gąsiorowski K., Marecik T., Szczurowski P., Zapała J., Wyszyńska-Pawelec G. (2021). Epidemiology of Primary Epithelial Salivary Gland Tumors in Southern Poland-A 26-Year, Clinicopathologic, Retrospective Analysis. J. Clin. Med..

[13] Aga M., Kondo S., Yamada K., Sawada-Kitamura S., Yagi-Nakanishi S., Endo K., Wakisaka N., Murono S., Kawano M., Yoshizaki T. (2013). Warthin’s tumor associated with IgG4-related disease. Auris Nasus Larynx.

[14] Aoki R., Tanaka T. (2024). Pathogenesis of Warthin’s Tumor: Neoplastic or Non-Neoplastic?. Cancers.

[15] Kadletz L., Grasl S., Perisanidis C., Grasl M.C., Erovic B.M. (2019). Rising incidences of Warthin’s tumors may be linked to obesity: A single-institutional experience. Eur. Arch. Otorhinolaryngol..

[16] World Obesity Federation (2020). Global trends in obesity. Obesity: Missing the 2025 Global Targets.

[17] OECD (2019). OECD Health Policy Studies. The Heavy Burden of Obesity: The Economics of Prevention.

[18] Hill J.H., Solt C., Foster M.T. (2018). Obesity associated disease risk: The role of inherent differences and location of adipose depots. Horm. Mol. Biol. Clin. Investig..

[19] Ostrowska L., Smarkusz-Zarzecka J., Gornowicz A., Lendzion K., Zyśk B., Pogodziński D. (2023). Analysis of Selected Salivary Adipokines and Cytokines in Patients with Obesity-A Pilot Study. Int. J. Mol. Sci..

[20] Kolb R., Sutterwala F.S., Zhang W. (2016). Obesity and cancer: Inflammation bridges the two. Curr. Opin. Pharmacol..

[21] Lehmann A.P., Nijakowski K., Swora-Cwynar E., Łuczak J., Czepulis N., Surdacka A. (2020). Characteristics of salivary inflammation in obesity. Pol. Arch. Intern. Med..

[22] Suba Z., Barabás J., Szabó G., Takács D., Ujpál M. (2005). Increased prevalence of diabetes and obesity in patients with salivary gland tumors. Diabetes Care.

[23] Leopard D., El-Hitti E., Puttasiddaiah P., Mcleod R., Owens D. (2022). Twenty-seven years of primary salivary gland carcinoma in Wales: An analysis of histological subtype and associated risk factors. J. Laryngol. Otol..

[24] Forrest J., Campbell P., Kreiger N., Sloan M. (2008). Salivary gland cancer: An exploratory analysis of dietary factors. Nutr. Cancer.

[25] Yaor M.A. (2010). The pattern of presentation of salivary gland tumors in Africa: A review of published reports. Ear Nose Throat J..

[26] Vuhahula E.A. (2004). Salivary gland tumors in Uganda: Clinical pathological study. Afr. Health Sci..

[27] Global Obesity Observatory. https://data.worldobesity.org/.

[28] Sadetzki S., Oberman B., Mandelzweig L., Chetrit A., Ben-Tal T., Jarus-Hakak A., Duvdevani S., Cardis E., Wolf M.S. (2008). Smoking and risk of parotid gland tumors: A nationwide case-control study. Cancer.

[29] Lewis P.D., Baxter P., Paul Griffiths A., Parry J.M., Skibinski D.O. (2000). Detection of damage to the mitochondrial genome in the oncocytic cells of Warthin’s tumour. J. Pathol..

[30] Larson N.I., Story M., Perry C.L., Neumark-Sztainer D., Hannan P.J. (2007). Are diet and physical activity patterns related to cigarette smoking in adolescents? Findings from Project EAT. Prev. Chronic Dis..

[31] Yang Y., Yang C., Lei Z., Rong H., Yu S., Wu H., Yang L., Lei Y., Liu W., Nie Y. (2021). Cigarette smoking exposure breaks the homeostasis of cholesterol and bile acid metabolism and induces gut microbiota dysbiosis in mice with different diets. Toxicology.

[32] Birch J., Gil J. (2020). Senescence and the SASP: Many therapeutic avenues. Genes Dev..

[33] Watanabe S., Kawamoto S., Ohtani N., Hara E. (2017). Impact of senescence-associated secretory phenotype and its potential as a therapeutic target for senescence-associated diseases. Cancer Sci..

[34] Lecot P., Alimirah F., Desprez P.Y., Campisi J., Wiley C. (2016). Context-dependent effects of cellular senescence in cancer development. Br. J. Cancer.

[35] Campisi J. (2013). Aging, cellular senescence, and cancer. Annu. Rev. Physiol..

[36] Quer M., Hernandez-Prera J.C., Silver C.E., Casasayas M., Simo R., Poorten V.V., Guntinas-Lichius O., Bradley P.J., Tong-Ng W., Rodrigo J.P. (2021). Current Trends and Controversies in the Management of Warthin Tumor of the Parotid Gland. Diagnostics.

[37] Do K., Kawana E., Tian S., Bigcas J.L. (2024). Treatment of Warthin’s Tumors of the Parotid Gland with Radiofrequency Ablation: A Systematic Review of the Current Literature. Ear Nose Throat J..

[38] Jin M., Fu J., Lu J., Xu W., Chi H., Wang X., Cong Z. (2019). Ultrasound-guided percutaneous microwave ablation of parotid gland adenolymphoma: A case report. Medicine.

[39] Tung Y.C., Luo S.D., Su Y.Y., Chen W.C., Chen H.L., Cheng K.L., Lin W.C. (2019). Evaluation of Outcomes following Radiofrequency Ablation for Treatment of Parotid Tail Warthin Tumors. J. Vasc. Interv. Radiol..

[40] Mamidi I.S., Lee E., Benito D.A., Li L., Goodman J.F., Thakkar P.G., Joshi A. (2021). Ultrasound-guided ethanol sclerotherapy for non-surgical treatment of Warthin’s tumor. Am. J. Otolaryngol..

[41] Schwalje A.T., Uzelac A., Ryan W.R. (2015). Growth rate characteristics of Warthin’s tumours of the parotid gland. Int. J. Oral. Maxillofac. Surg..

[42] Faur A.C., Sas I., Motoc A.G., Cornianu M., Zamfir C., Lazăr D.C., Folescu R. (2015). Ki-67 and p53 immunostaining assessment of proliferative activity in salivary tumors. Rom. J. Morphol. Embryol..

[43] Shou L., Chen X., Yang J., Jiang Y. (2022). Malignant transformation of Warthin’s tumor into squamous cell carcinoma: A case report. Oncol. Lett..

[44] Iyengar N.M., Gucalp A., Dannenberg A.J., Hudis C.A. (2016). Obesity and Cancer Mechanisms: Tumor Microenvironment and Inflammation. J. Clin. Oncol..

